# Multiplex bacterial polymerase chain reaction in a cohort of patients with pleural effusion

**DOI:** 10.1186/s12879-020-4793-6

**Published:** 2020-02-01

**Authors:** Léo Franchetti, Desiree M. Schumann, Michael Tamm, Kathleen Jahn, Daiana Stolz

**Affiliations:** Clinic of Pulmonary Medicine and Pulmonary Cell Research, University Hospital Basel, Petersgraben 4, 4031 Basel, CH Switzerland

**Keywords:** Multiplex PCR, Pleural empyema, Bacteria, Pathogen, Para-pneumonic effusion

## Abstract

**Background:**

The identification of the pathogens in pleural effusion has mainly relied on conventional bacterial culture or single species polymerase chain reaction (PCR), both with relatively low sensitivity. We investigated the efficacy of a commercially available multiplex bacterial PCR assay developed for pneumonia to identify the pathogens involved in pleural infection, particularly empyema.

**Methods:**

A prospective, monocentric, observational study including 194 patients with pleural effusion. Patients were evaluated based on imaging, laboratory values, pleura ultrasound and results of thoracentesis including conventional microbiology studies during hospitalisation. Multiplex bacterial PCR (Curetis Unyvero p55) was performed in batch and had no influence on therapeutic decisions.

**Results:**

Overall, there were 51/197 cases with transudate and 146/197 with exudate. In 42% (*n* = 90/214) there was a clinical suspicion of parapneumonic effusion and the final clinical diagnosis of empyema was made in 29% (*n* = 61/214) of all cases. The most common microorganisms identified in the cases diagnosed with empyema were anaerobes [31] followed by gram-positive cocci [10] and gram-negative rods [4]. The multiplex PCR assay identified more of the pathogens on the panel than the conventional methods (23.3% (7/30) vs. 6.7% (2/30), *p* = 0.008).

**Conclusion:**

The multiplex PCR-based assay had a higher sensitivity and specificity than conventional microbiology when only the pathogens on the pneumonia panel were taken into account. A dedicated pleural empyema multiplex PCR panel including anaerobes would be needed to cover most common pathogens involved in pleural infection.

## Background

Microbial diagnosis and the identification of the pathogens causing pleural infection have generally relied on conventional bacterial culture [1]. Due to the increase in the complexity of the pathogens and the increased use of antibiotic pre-treatment, the sensitivity of the conventional bacterial culture has decreased [2, 3]. Also, culture of pleural fluid is difficult, particularly with the use of broad-spectrum antibiotics, which can hinder bacterial growth. The highest yield of 60% can be achieved with inoculation in standard blood culture bottles [4]. The high rate of culture-negative samples thus complicates clinical care and often precludes streamlining to selective antibiotics.

With the advancement in technology, molecular techniques have been implemented for accurate pathogen identification in diagnostic microbiology. Broad-range 16S rRNA gene polymerase chain reaction (PCR) is often used to detect and identify all bacterial pathogens in primary sterile clinical specimens, mostly in cases where bacterial infection is suspected, but where cultures are negative. PCR using broad-range 16S rRNA gene primers has been used to determine the aetiology of pleural empyema and offers a relatively high detection rate of up to 85% in a single assay, allowing the rapid introduction of targeted antibiotic therapy [3, 5–7]. In previous studies, multiplex PCR methods in different matrices (blood, sputum, throat or nasopharyngeal swabs) have proven to be useful in diagnosing respiratory tract infections and decreasing the financial costs [8]. They have also shown a better sensitivity [9, 10], especially in children [11, 12]. They also seem to be effective for reducing the incidence of empyema [12]. However, some studies have shown limitations to the sensitivity of the PCR [13].

Whether a multiplex bacterial PCR focusing on pathogens commonly encountered in pneumonia improves the diagnosis and management of patients with pleural effusion remains unexplored. Thus, the objective of this study was to investigate the diagnostic performance of a commercially available multiplex bacterial PCR-based assay in the pleural fluid of patients with pleura effusion. We hypothesize that the molecular diagnostic technique improves the diagnostic yield of conventional culture for the detection of pathogens in pleural effusion.

## Methods

This was a prospective, non-interventional study conducted in the University Hospital Basel, Switzerland. This study was conducted in accordance with the amended Declaration of Helsinki. An independent ethics committee, Ethikkommission Beider Basel, approved the study (EKBB 120/10) and the subjects provided written informed consent.

### Study design

All hospitalized adult patients with pleural effusion referred to the Clinic of Pneumology at the University Hospital Basel, Switzerland during a period of 48 months were considered for the study. Inclusion criteria were: a) age > 18 years; b) more than “minimal” pleural effusion, i.e. an effusion requiring diagnostic or therapeutic procedure; c) informed consent signed by the patient. The first pleural effusion sampling was used for analysis in cases where pleural effusion sampling from the same patient occurred more than once during a single hospital visit. Immunosuppression was defined as manifested AIDS, chemotherapy, status after solid organ transplantation, status after haematological stem cell transplantation, progressive uncontrolled malignancy or neutropenia (< 500*10^9^/L).

### Diagnostic work-up

Pleural effusion specimens were collected and examined routinely. Gram and other appropriate stains and cultures for bacteria, mycobacteria, and fungi were performed according to standard procedures [14]. Cell differentiation in the pleural fluid was reported as absolute numbers and as a percentage of the total cell count.

### Conventional methods

Forty microliters of vortexed pleural fluid were added to culture plates including Columbia sheep blood agar, Colistin Nalidixic Acid blood agar for Gram positive bacteria, Haemophilus chocolate agar and MacConkey for Gram negatives [14]. These plates were incubated for 2 days at 36 °C. In the case of additional investigations such as for the *Legionella* spp. or moulds, specific selective plates for these pathogens were inoculated additionally. The incubation times and conditions varied according to specific pathogens. Isolates were identified by matrix-assisted laser desorption/ionization-time-of-flight (MALDI-TOF), mass spectrometry (MALDI BioTyper, Bruker Daltonics, Bremen, Germany), biochemical profiling or 16S rDNA sequencing.

All samples were cultured at the time of hospitalisation and used for the diagnoses.

### Curetis Unyvero PHN assay

Molecular analysis of pleural fluid was performed in batch on stored samples using the multiplex PCR-based assay testing system from Curetis AG (Holzgerlingen, Germany), according to the manufacturers’ instructions. This assay panel targets specific genes for the following 20 bacteria: *Staphylococcus aureus*, *Streptococcus pneumoniae*, *Enterobacteriaceae*, *Citrobacter freundii*, *Escherichia coli*, *Enterobacter cloacae* complex, *Enterobacter aerogenes*, *Proteus* spp., *Klebsiella pneumoniae*, *Klebsiella oxytoca*, *Klebsiella variicola*, *Serratia marcescens*, *Morganella morganii*, *Moraxella catarrhalis*, *Pseudomonas aeruginosa*, *Acinetobacter baumannii* complex, *Stenotrophomonas maltophilia*, *Legionella pneumophila*, *Haemophilus influenza*, *Mycoplasma pneumoniae* and one fungus: *Pneumocystis jirovecii*. It is also able to recognize 22 genetic markers of resistance including β-lactam, macrolide, fluoroquinolone and Carbapenem resistance.

Time estimated for sample preparation, DNA extraction and purification, amplification and specific detection is approximately 4 h. Operators of the assay and all other microbiological methods were unaware of the results and did not participate in any data analysis.

Results of the commercial multiplex PCR did not impact patient management. The samples were stored at − 20 °C immediately after extraction from the body. The samples were then thawed at 4 °C for approximately 3 h after which it was aliquoted and subsequently stored at − 80 °C.

### Diagnostic criteria and data collection

Data were collected at the time of pleural fluid sampling and included demographics, comorbidities, laboratory findings, radiologic results, medication history, final diagnosis (based on all available clinical and laboratory results) and clinical outcome. Samples were dichotomized in transudate and exudate according to the Light criteria [15, 16]. Empyema was defined as a positive Gram stain or bacterial culture, the presence of pus (macroscopic purulence) in the pleural space or pleural fluid pH < 7.2 with clinical evidence of infection [17].

### Statistical analysis

Numerical results were expressed as mean (standard deviation) or median (interquartile range) unless otherwise stated. Differences in dichotomous variables were evaluated using the Chi-square test. All other continuously non-normally distributed parameters were evaluated using the non-parametric Mann–Whitney U test. Reference standards used to calculate the diagnostic yield (sensitivity and specificity) of the bacterial multiplex PCR analyses were threefold: conventional culture, clinical diagnosis of empyema as described by the attending physician in the final hospitalisation report and pleural effusion pH ≤ 7.2. A *p*-value of < 0.05 was considered significant. No imputation was made when data were missing. Data analyses were conducted according to the Statistical Package for Social Sciences (SPSS, version 22 for Windows) program.

## Results

**Fig. 1:**
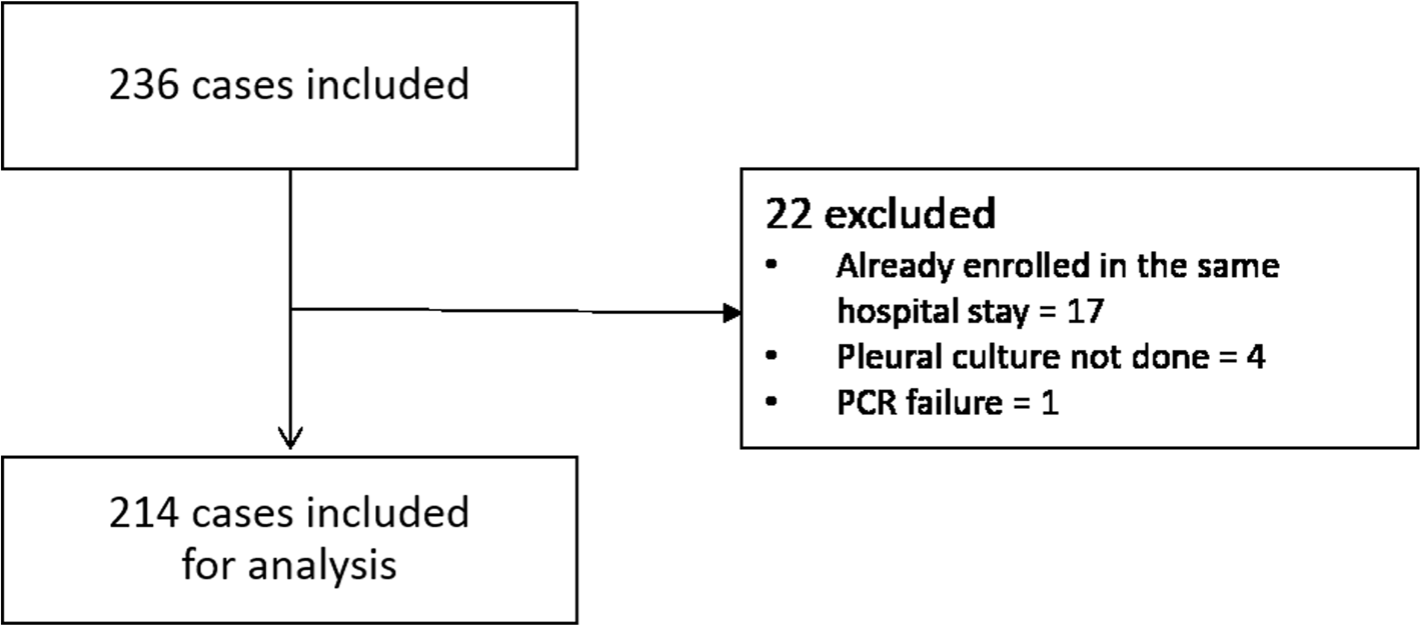
Flow chart depicting patient cohort selection criteria

A total of 194 patients with 214 visits were included in the study (Fig. 1). More than half of the participants were male and the average age was 68.8 ± 15.5 years (Table 1). Twenty-two percent of the patients were current smokers and the most common symptom at admission was cough. The 30-day mortality was 9.8% (*n* = 19/194) and the 90-day mortality was 13.9% (*n* = 27/194). The most prevalent antibiotic class was penicillins (93/128) followed by cephalosporins (34/128) and carbapenems (20/128). Antibiotics with anaerobic coverage were given as treatment to 105/128 cases. All patients underwent imaging studies with a thoracic ultrasound performed in nearly 90% of the cases. Various radiological investigations were performed to diagnose the patient (Table 2).

**Table 1:** Basic characteristics of the study participants with pleural effusion, *n* = 194; cases = 214

	*N* = 194	Missing data
Age, y	68.8 ± 15.5	0
Male	112 (58)	0
unadjusted Charlson Score	4.8 ± 2.7	0
ASA Score	3.1 ± 0.44	0
Length of hospital stay, days	16.0 (7.0;27.5)	0
Smoking status		10
Current smoker	42 (22)	
Non-smoker	61 (31)	
Immune status		
Immunosuppressed	40 (18.6)	1
Signs and symptoms		2
Cough	91 (43)	
Sputum	56 (26)	
Fever	58 (27)	
Chest pain	53 (25)	
Blood parameters		
Performed	206 (96)	8
Leucocytes (*10^9^/l)	10.4 ± 7.6	0
Neutrophils (*10^9^/l)	7.9 ± 5.5	32
C-reactive protein (mg/l)	118.1 ± 109.8	3
Lactate dehydrogenase (U/l)	287.4 ± 208.9	6
Pleural fluid parameters		
Performed	214 (100)	
Transudate	51 (26)	7
Exudate	146 (74)	7
Protein (g/l)	34.2 ± 11.6	7
Glucose (mmol/l)	5.6 ± 2.9	26
pH	7.3 ± 0.23	35
pH ≤ 7.2	37 (21)	35
Lactate dehydrogenase (U/l)	1047.9 ± 3895.3	20
Anti-infective therapy before sampling	130 (61)	0
Antibiotics	128 (60)	0
Anti-fungal	11 (5)	0
Anti-viral	10 (5)	0
Duration of antibiotic therapy		
Before sampling	1 [0;7]	1
After sampling	8 [0;21]	0
Total	14 [0;34]	1
Final clinical diagnosis		
Empyema	61 (29)	0
Therapeutic regime		
Conservative	175 (82)	0
Operative	39 (18)	0
Drainage	158 (74)	0
Medical Thoracoscopy	83 (39)	0
Outcomes		
In-house mortality within 30 days of sampling	18 (9.3)	0
Re-hospitalisation within 90 days of discharge	68 (35)	0
Operation within 60 days of sampling	57 (27)	0

Median (IQR) or mean (standard deviation), n (%)

**Table 2:** Radiological investigations of the patients included in the study

	Patient number = 194; cases = 214
X-Ray results	
Performed	128 (60)
Effusion	126 (98)
Meniscus	62 (48)
Infiltrate	39 (30)
Suspicion of empyema	4 (3)
CT-scan findings	
Performed	139 (65)
Effusion	139 (100)
Infiltrate	52 (37)
Nodule/Tumor	49 (35)
Enhancement	20 (14)
Suspicion of empyema	18 (13)
Ultrasound findings	
Performed	189 (88)
Hyperechogenicity	19 (10)
Septa	42 (22)
Massive organisation	35 (19)
single category	73 (34)
Macroscopic bronchoscopy findings	
Performed	73 (34)
No pathological finding	24 (33)
Non-purulent secretion	23 (32)
Erythema of the mucosa	20 (27)
Purulent secretion	17 (23)
Endobronchial tumour	8 (11)
Bloody secretion	5 (7)

There were 51/197 (26%) samples classified as transudate and 146/197 (74%) were classified as exudate. Among the exudates, 18/146 (12%) samples were positive for pathogens when using conventional methods compared to 13/146 (8.9%) positive samples when using the multiplex PCR-based assay (*Curetis Unyvero P55 assay*). Three of the pathogens identified using conventional methods were on the *Curetis Unyvero P55* panel (Table 3).

**Table 3:** Pathogens and how often the pathogens were identified using conventional methods compared to the multiplex PCR-based assay

Pathogen	Conventional method positive	Multiplex PCR-based assay positive	
*Citrobacter freundii*		1	Gram-negative
*Enterobacter cloacae*	1		Gram-negative
*Proteus* spp.		1	Gram-negative
*Pseudomonas aeruginosa*		1	Gram-negative
*Staphylococcus aureus*	1	2	Gram-positive
*Stenotrophomonas maltophilia*		5	Gram-negative
*Streptococcus pneumoniae*	1	5	Gram-positive
Pathogens not on the Curetis p55 panel			
*Bacteroides fragilis*	1		Anaerobic
*Campylobacter* species	2		Anaerobic
*Eikenella corrodens*	2		Anaerobic
*Enterococcus faecium*	1		Gram-positive
*Finereferenceia magna*	1		Anaerobic
*Fusobacterium* nucleatum	4		Anaerobic
*Lactobacillus*	1		Anaerobic
*Micrococcus luteus*	1		Gram-positive
*Mycobacterium avium*	1		Gram-positive
*Parvimonas micra*	2		Anaerobic
*Prevotella* species	4		Anaerobic
*Propionibacterium acnes*	1		Anaerobic
*Staphylococcus capitis*	1		Gram-positive
*Staphylococcus hominis*	1		Gram-positive
*Streptococcus agalactiae*	1		Gram-positive
*Streptococcus anginosus*	5		Anaerobic
*Streptococcus constellatus*	1		Anaerobic
*Streptococcus intermedius*	2		Anaerobic
*Streptococcus milleri*	4		Anaerobic
*Streptococcus mitis*	1		Anaerobic
*Streptococcus sanguinis*	1		Anaerobic
*Veillonella* species	1		Anaerobic

When only taking into account the 21 pathogens included in the *Curetis Unyvero P55* panel, more samples were positive for pathogens using the multiplex PCR-based assay (*n* = 15/214) compared to conventional methods (*n* = 3/214, *p* < 0.001, Table 3). The sensitivity of the multiplex PCR-based assay was 67% and specificity was 94% when using the conventional method as a reference. (Table 4). When considering all pathogens, i.e. also pathogens not included in the panel, the sensitivity of the multiplex PCR-based assay decreased (Table 4).

**Table 4:** Diagnostic performance of conventional methods and the multiplex PCR-based assay and using conventional methods, final clinical diagnosis of empyema, empyema and pleura fluid ≤7.2 as reference-standard

1. All cases – Conventional methods as reference-standard	Sensitivity (%)	Specificity (%)	Positive predictive value (%)
Multiplex PCR-based assay vs. all pathogens	12	93.5	20
Multiplex PCR-based assay vs. only panel pathogens	67	93.7	13
2. Using cases with final clinical diagnosis of empyema - conventional methods as reference-standard	Sensitivity	Specificity	
Multiplex PCR-based assay vs. all pathogens	15.8	80	27
Multiplex PCR-based assay vs. only panel pathogens	100	84.2	18
3. Using cases with or without empyema diagnosis as reference-standard	Sensitivity	Specificity	
Conventional methods (all pathogens)	32.8	96.1	77
Conventional methods only panel pathogens	3.3	99.3	67
Multiplex PCR-based assay	18.6	97.3	73
4. Using cases with or without pleural fluid pH ≤ 7.2 as reference standard	Sensitivity	Specificity	
Conventional methods (all pathogens)	37.8	95.8	70
Conventional methods only panel pathogens	2.7	99.3	100
Multiplex PCR-based assay	13.5	97.1	56

There was a suspicion of empyema based on the ultrasound findings in 35/189 (18.5%) cases and from those, 30 cases (85.7%) were diagnosed with empyema (16 hospital-acquired and 14 community-acquired pneumonia). Of all the cases with suspicion of empyema, 47/79 (22.0%) were diagnosed with empyema and, of the cases with no suspicion of empyema at admission, 14/135 (10.4%) had a final diagnosis of empyema. In total, 29% (*n* = 61/214) of the cases had a final clinical diagnosis of empyema. The cases with empyema were significantly younger, with lower blood lactate dehydrogenase values but higher pleural fluid lactate dehydrogenase levels, longer antibiotic treatment before sampling and longer hospital stays compared to cases without empyema (Table 5). As expected, pleural fluid pH and glucose were significantly lower in cases with empyema compared to cases without empyema. There was no association between mortality (Exp β =1.394, 95% CI 0.551–3.530; *p* = 0.483) or immune status (Exp β = 0.676, 95% CI 0.300–1.519; *p* = 0.343) and the empyema diagnosis.

**Table 5:** Patients with a diagnosis of no empyema compared to patients with a final diagnosis of empyema

	No Empyema	Empyema	*p*-value
Age, y	70.4 ± 15.4	65.0 ± 15.2	**0.012**
Length of stay, days	13 (4.0;22.0)	25.0 (15.0;35.5)	**< 0.001**
unadjusted Charlson score	4.9 ± 2.6	4.7 ± 3.1	0.572
ASA score	3.1 ± 0.46	3.0 ± 0.4	0.668
Smoking status			**0.006**
Current smoker	25 (17)	23 (38)	
Non-smoker	49 (34)	16 (27)	
Blood parameters			
Leucocytes (*10^9^/l)	9.2 ± 7.4	13.3 ± 7.1	**< 0.001**
Neutrophils (*10^9^/l)	6.2 ± 3.5	11.3 ± 7.1	**< 0.001**
C-reactive protein (mg/l)	81.3 ± 83.4	203.7 ± 116.6	**< 0.001**
Lactate dehydrogenase (U/l)	315.8 ± 229.4	222.8 ± 132.6	**< 0.001**
Pleural fluid parameters			
Protein (g/l)	33.6 ± 11.3	36.1 ± 12.2	0.074
Glucose (mmol/l)	6.1 ± 2.7	4.3 ± 3.2	**< 0.001**
pH	7.4 ± 0.11	7.1 ± 0.3	**< 0.001**
LDH (U/l)	439.5 ± 637.5	3339.3 ± 7558.9	**< 0.001**
Antibiotic duration (days)			
Before sampling	0 (0;6)	4.0 (1.0;9.0)	**< 0.001**
After sampling	5.0 (0;10)	26.0 (15.5;37.5)	**< 0.001**
Total antibiotic treatment	7.0 (0;15)	31.0 (20;44)	**< 0.001**
Patient outcome			
30-day mortality	12 (9)	7 (12)	0.598

Boldface *p*-values are significant, that is *p* < 0.05

When using final clinical diagnosis of empyema as the reference-standard, the multiplex PCR-based assay had a lower sensitivity but slightly higher specificity than conventional methods (Table 4). However, when restricting the analyses to the 21 pathogens available on the panel, the sensitivity of the conventional methods decreased to 3.3%.

Four samples positive for pathogens (*Stenotrophomonas maltophilia*, *Citrobacter freundii*, *Pseudomonas aeruginosa* and *Proteus* spp.) using the multiplex PCR and six samples positive for pathogens (*Staphylococcus capitis*, *Staphylococcus hominis*, *Mycobacterium avium*, *Enterobacter cloacae*, *Campylobacter concisus*, *Veillonella* spp., *Propionibacterium acnes* and *Micrococcus luteus*) using conventional methods were considered to be negative for empyema in the final clinical diagnosis.

The most common microorganisms identified by conventional methods and the multiplex PCR assay in the cases diagnosed with empyema were anaerobes (31/45) followed by gram-positive cocci (10/45) and gram-negative rods (4/45). About one third of all the cases (*n* = 9/26, 35%) proved to be multi-bacterial when using conventional methods (Table 6).

**Table 6:** Pathogens identified in multi-bacterial samples

	Pathogens
Sample 1	*Streptococcus anginosus, Prevotella* spp*., Parvimonas micra*
Sample 2	*Lactobacillus, Streptococcus intermedius*
Sample 3	*Streptococcus constellatus, Parvimonas micra, Prevotella* spp*., Campylobacter* spp*., Fusobacterium* spp*.*
Sample 4	*Streptococcus milleri, Eikenella corrodens*
Sample 5	*Eikenella corrodens, Fusobacterium* spp*., Prevotella oris*
Sample 6	*Campylobacter concisus, Veillonella* spp*.*
Sample 7	*Staphylococcus capitis, Staphylococcus hominis*
Sample 8	*Streptococcus agalactiae, Streptococcus anginosus, Finereferenceia magna, Prevotella* spp*., Fusobacterium* spp*.*
Sample 9	*Streptococcus milleri, Fusobacterium nucleatum*

In the cases with a final diagnosis of empyema, there was a significant association between antibiotic therapy before sampling and conventional culture results with a 10.5x increase in the odds of a negative culture result [Exp(B) = 10.5, 95% CI 1.933–57.029; *p* = 0.006]. Also between duration of antibiotic therapy before sampling [Exp(B) = 0.877 95% CI 0.774–0.993; *p* = 0.039] and conventional culture results. There was no association between antibiotic therapy before sampling (Exp(B) = 1.556, 95% CI 0.269–8.995; *p* = 0.622), the duration of antibiotic therapy before sampling (Exp(B) = 1.008, 95% CI 0.931–1.092; *p* = 0.836) and the multiplex PCR assay result.

## Discussion

Pleural infection is a common complication in pneumonia and linked with high mortality [18, 19]. Early and reliable detection of the causative organism is beneficial in establishing the correct pathogen-specific treatment, which may lead to decreased morbidity and mortality. However, due to the low sensitivity of conventional cultures [1], the diagnosis and management of pleural infection remains challenging [2, 3].

In this study we assessed the diagnostic accuracy of a commercial multiplex bacterial PCR for the detection of pathogens in pleural effusion compared with conventional methods in a large group of patients with pleural effusion. We found that the multiplex PCR had a better sensitivity and specificity than conventional methods if the analysis is restricted to microorganisms included on the panel. Indeed, when using the clinical final diagnosis of empyema as a reference, the multiplex PCR-based assay had sensitivity six-times higher than that of the conventional microbiologic methods. However, only a minority of the samples and a small percentage of the pathogens diagnosed by conventional methods were included in this panel including bacteria commonly involved in pneumonia. Considering that empyema is a common complication of pneumonia, it would be reasonable to suggest that the pathogens involved in empyema are similar to those found in pneumonia. Based on pathogens identified for pleural fluid in various other publications [2, 3, 5, 6, 13, 20–22], at least 50% of the known pathogens were available on the multiplex PCR-based assay. Yet, only 7 (24%) of the 29 pathogens identified by either conventional or molecular methods in this study were included on the panel of this multiplex bacterial PCR for pneumonia.

Most of the pathogens detected in the patients diagnosed with empyema were anaerobes (69%) followed by gram-positive cocci (22%). These figures were unexpected and differ from previous reports suggesting that organisms related to community-acquired pneumonia, such as *S. pneumonia*, *H. influenza*, *S. aureus, K.Pneumoniae,* are the most common causes of empyema [2, 3, 5, 13, 20, 23]. A more recent paper by Johansson et al. [24], however, had similar results to our study, in that they found an anaerobe presence of 40%. Thus, empyema might require anaerobe coverage more often than commonly reported.

*Streptococcus pneumoniae*, the most common pathogen causing parapneumonic effusions [2, 3, 10, 25] was identified in 5 samples using the multiplex PCR-based assay and only once when using conventional methods. Conventional methods identified only one *Stenotrophomonas maltophilia* sample compared to the multiplex PCR-based assay that identified it in five samples. Although *Stenotrophomonas maltophilia* is a gram-negative pathogen known to cause many severe infections and can result in mortality in approximately 14.6 to 42.5% of patients [26–28], pleural infection associated with this organism is rare [28]. *Stenotrophomonas maltophilia* is thought to cause nosocomial infections mainly in immunocompromised patients [28], but in our study, only one patient identified with this pathogen was immunocompromised and four of the five cases positive for *Stenotrophomonas maltophilia* had a final diagnosis of empyema. It is plausible that the molecular diagnostic technique could improve the recognition of this pathogen, which is associated with a particularly high mortality rate.

In 85% (52/61) of the cases diagnosed with empyema, antibiotics had been administered before sampling. As found previously [29], we found no association between the multiplex PCR-based assay and prior administration of antibiotics or duration of antibiotic use before sampling in the cases diagnosed with empyema. However, antibiotic use before sampling and the duration of antibiotic use before sampling was associated with more bacterial negative results when using the conventional method in the cases diagnosed with empyema.

When using the final clinical diagnosis of empyema (yes or no) as reference-standard, the multiplex PCR-based assay had a sensitivity of 18.6% and a specificity of 97.3%. When restricting the analysis to pathogens included in the panel, conventional methods had a sensitivity of 3.3%. Twenty-two pathogens were detected by conventional methods and were not on the multiplex PCR panel and when these pathogens were included in the analysis, conventional methods had a sensitivity of 32.8%.

The diagnostic accuracy of this multiplex bacterial panel focusing on pneumonia pathogens for pleural effusion was disappointingly low and highlighted the fact that before introduction in clinical practice, any diagnostic approach should be evaluated in a clinical study. It was apparent that a dedicated panel including anaerobes would be necessary to increase the diagnostic yield of a molecular technique aimed at diagnosing pleural infection.

In this study, we evaluated the diagnostic yield of conventional culture and a molecular technique in pleural effusions in a well-characterized, considerable sized cohort of patients. It is important to take into consideration that a bacterial multiplex PCR is limited its diagnostic yield due to the finite number of in-panel organisms. In addition, specificity issues regarding Enterobacter spp., *H. influenzae* and *S. pneumoniae* in comparison to culture-based methods have been previously reported for the molecular diagnostic test prototype [30]. Unlike others [30] with relevant technical failure rates when analysing respiratory secretions, the multiplex PCR assay had a 2.3% (5/214) failure rate in the current study when analysing pleural fluid.

Amongst its limitations, one has to underline the fact that the molecular diagnostics were performed on frozen material which may have affected the outcome of the analysis. Fernández-Soto P et al. found that less deoxyribonucleic acid (DNA) is extracted from urine frozen and stored for an extended time period compared to fresh urine [31]. Cushwa and Medrano found differences in DNA extraction from blood, based on length of storage time and storage temperature [32]. Thus, the sensitivity of the method may have been negatively impacted by the storage of the samples. However, the main obstacle for the detection of the pathogens by the PCR was the fact that they were not included in the spectrum of the panel for pneumonia. Thus, an increase in DNA amount was not expected to positively increase the sensitivity of the assay. Further, we had not used any further method to confirm the presence or absence of bacteria in the pleural effusion. This was a natural limitation as currently no diagnostic technique would be able to identify infection in this setting [33]. Nevertheless, the clinical final diagnosis had been additionally used as a reference-standard to identify infection. Recently, next-generation sequencing (NGS) was used to identify pathogens in empyema and 385 bacterial detections were made, compared to 38 by culture and 87 by 16S rRNA gene sequencing [34]. The authors concluded that a subgroup of patients with empyema have pathogens more resembling a brain abscess than pneumonia, ie. anaerobic pathogens and that this subgroup should be seen as primary empyema. With this new technique many more bacterial detections were made, but the conclusion that anaerobes are important in empyema remains the same as the conclusion we have drawn in our study. It would be interesting to compare sensitivity and specificity as well as cost-effectiveness between a multiplex PCR-based assay and NGS.

We were unable to determine the clinical implications of the commercial multiplex PCR-based assay on the treatment and outcome of the patients. Thus, further studies are necessary to evaluate whether a molecular diagnostic technique can be helpful in optimizing treatment of pleural infections. We believe that our results are generalizable, in that anaerobes may be important in empyema, but the exact species may differ between regions.

## Conclusion

In conclusion, most causative organisms were not included in the pneumonia panel. Thus, a dedicated pleural empyema multiplex PCR including anaerobes is required in pleural infection. The multiplex bacterial PCR assay had a higher sensitivity and specificity than conventional microbiology to diagnose bacterial infection in pleural effusion when only pathogens included on the panel were taken into account.

## Data Availability

The datasets used and/or analysed during the current study are available from the corresponding author on reasonable request.

## Abbreviations

DNA: Deoxyribonucleic acid
IQR: Interquartile range
PCR: Polymerase chain reaction
rRNA: Ribosomal ribonucleic acid
spp.: Species
